# Supplementary figures and images for: Cryo-EM reveals the conformational epitope of human monoclonal antibody PAM1.4 broadly reacting with polymorphic malarial protein VAR2CSA
Source: PLoS Pathog. 2022 Nov 16;18(11):e1010924. doi: 10.1371/journal.ppat.1010924 (PMC9668162; doi:10.1371/journal.ppat.1010924)

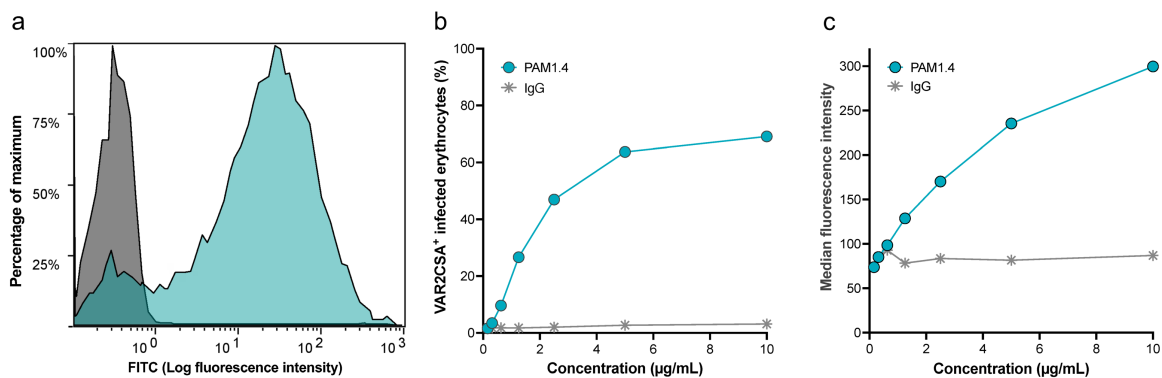

**S1 Fig. Reactivity of PAM1.4 to native VAR2CSA expressed on the surface of infected erythrocytes.**

Supplement: S1 Fig — (a) Representative flow cytometry histogram of FCR3 VAR2CSA in the presence of 10μg/ml PAM1.4 IgG (cyan) and 10μg/ml IgG isotype control (gray). (b,c) Erythrocytes infected by FCR3 VAR2CSA were incubated with PAM1.4 (seven two-fold serial dilutions from 0.16 to 10μg/ml). The percentage of FITC-positive cells (infected erythrocytes binding PAM1.4 or IgG isotype control) and the median fluorescence intensity were estimated and used to create dose-response curves. (PDF) [file ppat.1010924.s001.pdf]

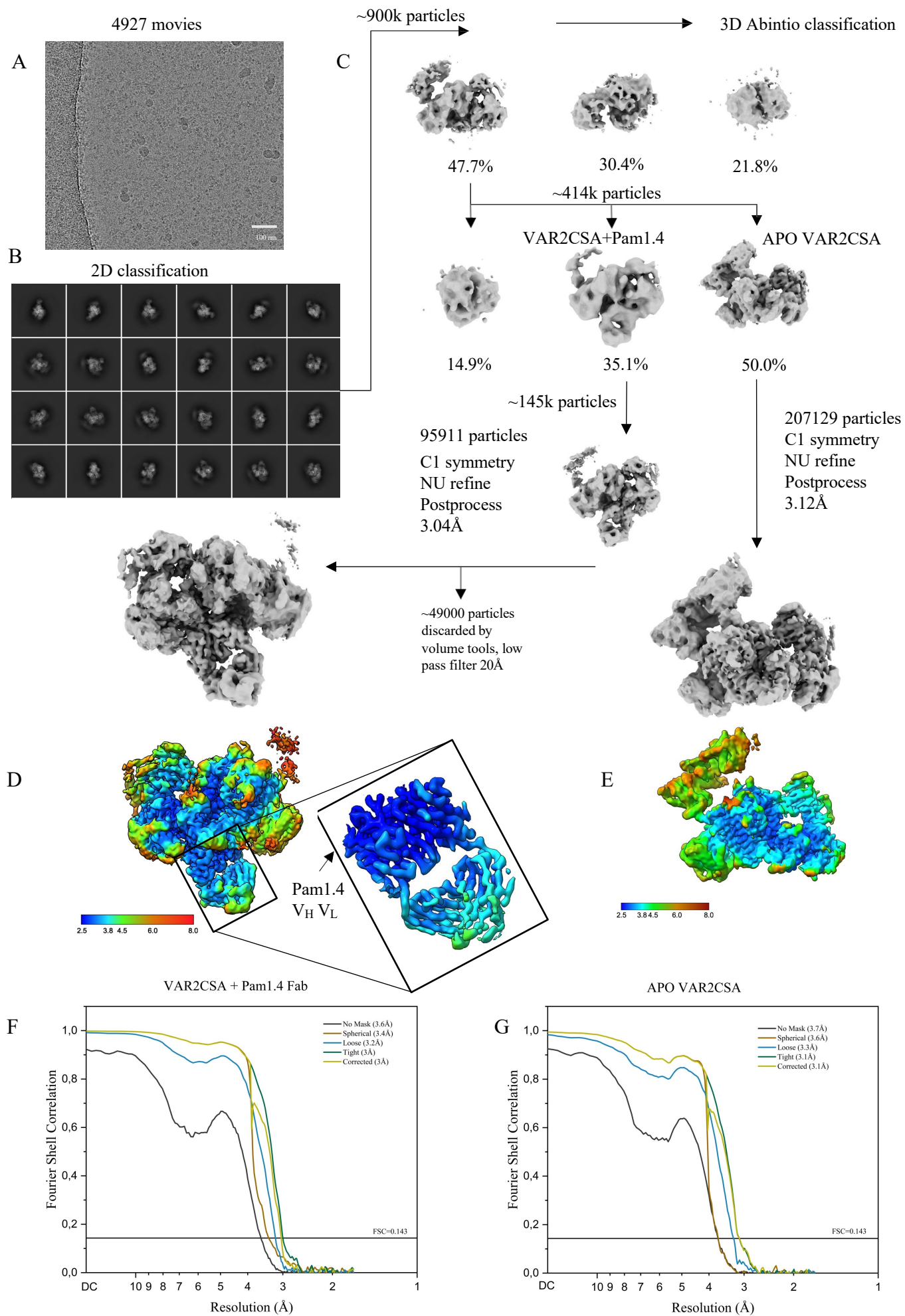

**S3 Fig. CryoEM workflow on determining VAR2CSA PAM 1.4 Fab structure.**

Supplement: S3 Fig — (a) Representative micrograph of the CryoEM grid used for structure determination. (b) Representative 2D class averages with box size of 44nm. (c) Flow chart of the data processing. Detailed method flow is described in the method section and S1 Table. (d) Final map of VAR2CSA PAM 1.4 Fab complex colored based on calculated resolution. Pam 1.4 Fab is magnified to highlight the resolution at the binding region of the Fab (Paratope). (e) Final map of APO VAR2CSA colored based on resolution. (f,g) Gold Standard Fourier Shell Correlation (FSC) curve of VAR2CSA PAM 1.4 (3.04 Å) and APO VAR2CSA (3.12 Å) respectively. (PDF) [file ppat.1010924.s003.pdf]

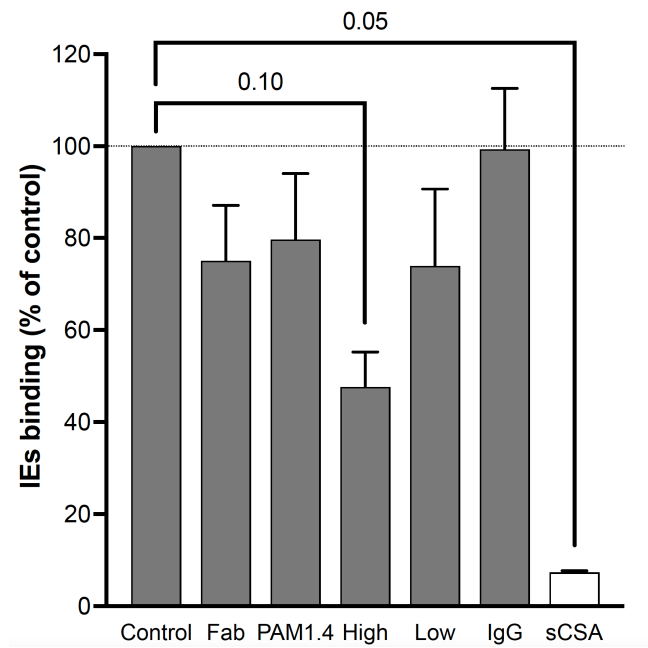

**S6 Fig. PAM1.4 does not inhibit the infected erythrocytes adhesion to CSA.**

Supplement: S6 Fig — Percentage of FCR3 VAR2CSA infected erythrocytes binding to CSA in the presence of 100 μg/ml antibodies or 500 μg/ml soluble CSA (sCSA). Control, absence of antibodies; Fab, PAM1.4 Fab fragment; PAM1.4, PAM1.4 whole IgG; high and low, corresponds to total IgG purified from a pool of plasma with high and low levels of anti-VAR2CSA, respectively; IgG, IgG isotype control. Median values ± 95% CI from two independent experiments and P values using Kruskal-Wallis test followed by Dunn’s multiple comparisons test are shown. (PDF) [file ppat.1010924.s006.pdf]

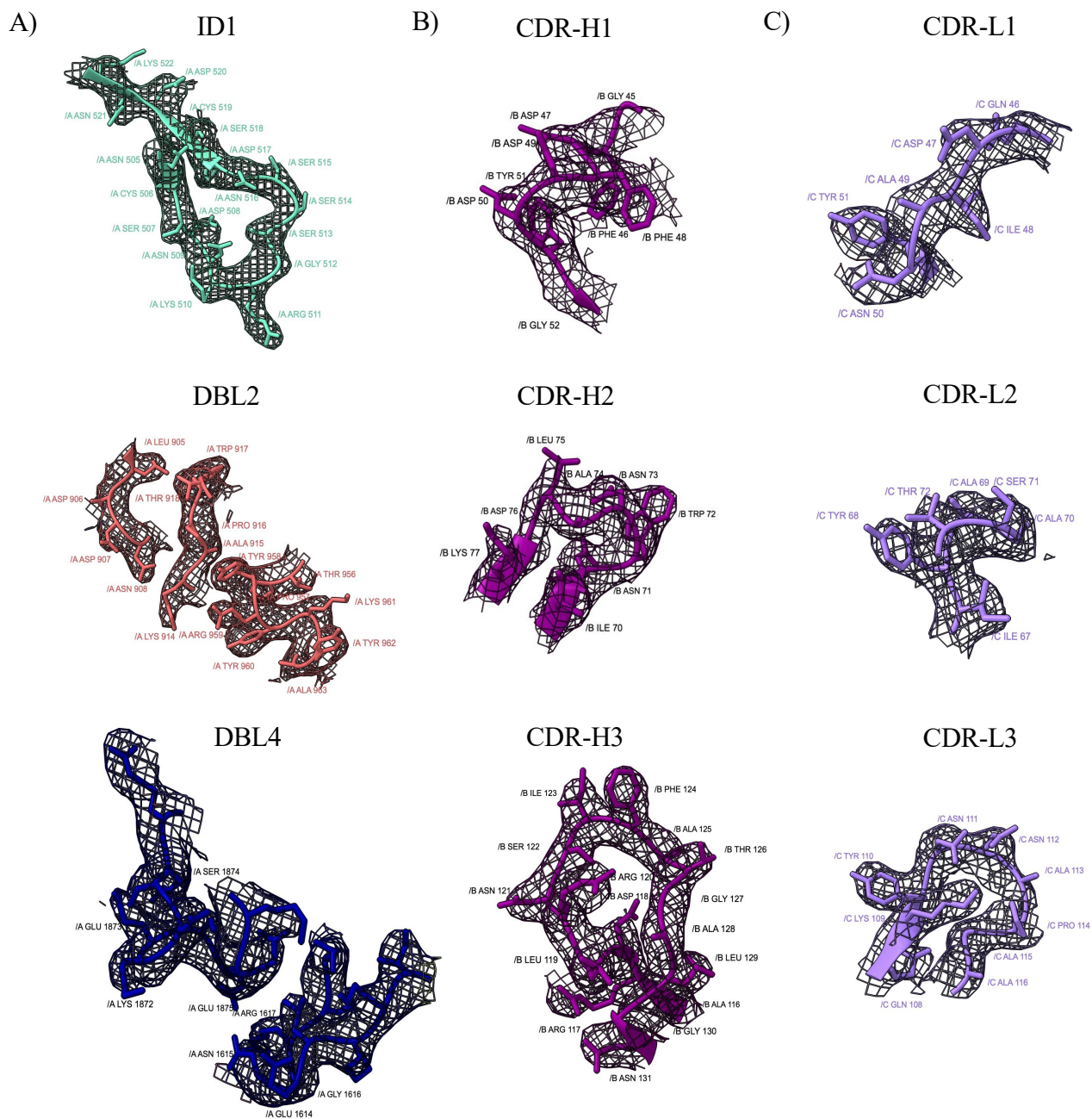

**S7 Fig. Quality of the cryoEM density.**

Supplement: S7 Fig — (a) ID1 loop: N505 –K522; DBL2: L905 –N908, K914 –W917, Y958 –A963; DBL4: E1614 –R1617, K1872 –E1875 (b) Heavy chain CDR loops, CDR–H1: D47 –G52; CDR–H2: I70 –K77; CDR–H3: R117 –N131. (c) Light chain CDR loops, CDR–L1: E46 –N50; CDR–L2: I67 –A70; CDR–L3: Q108 –A116. The volume surface is zoned at 2.5Å in UCSF ChimeraX for all the regions. (PDF) [file ppat.1010924.s007.pdf]

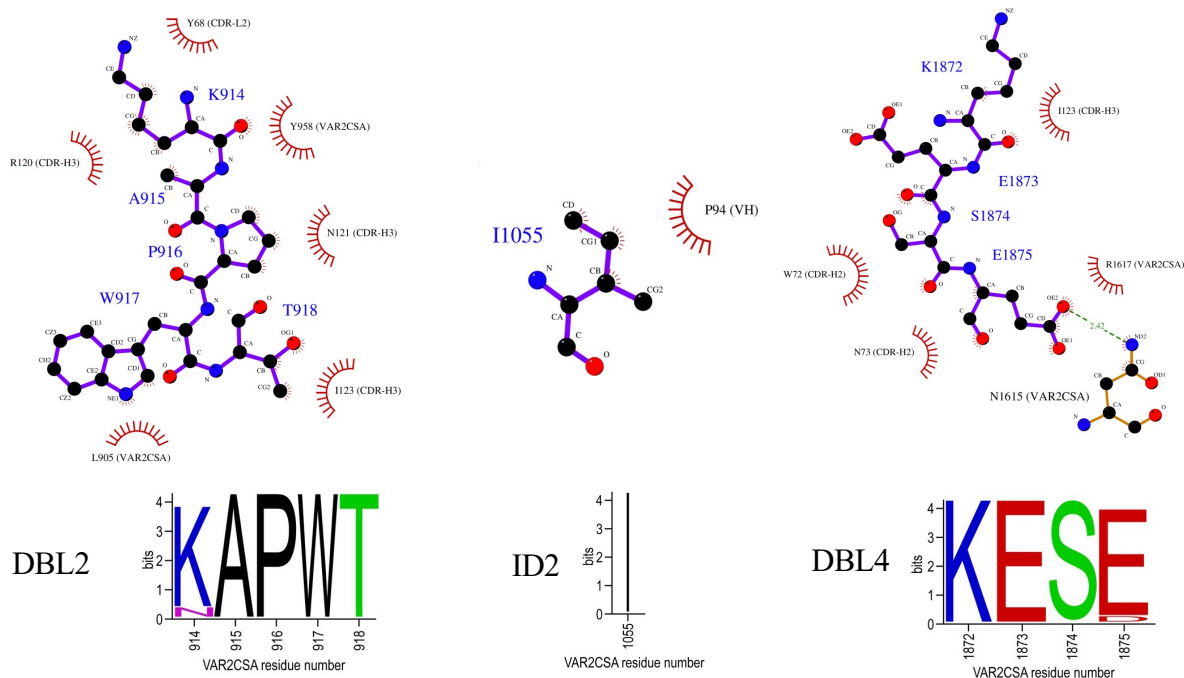

**S8 Fig. Epitopes of PAM 1.4 Fab interacting by hydrophobic contacts.**

Supplement: S8 Fig — Binding plot analysis is done using Ligplot+ v.2.7 as shown in Figs 4 and 5. Hydrophobic interaction is shown as red spokes radiating from the residues involved in the interaction. DBL2 and DBL4 are involed in binding with CDR–L2, H2 and H3. Non-CDR residue P94 of heavy chain interacts with ID2 residue I1055. Weblogo is used to represent sequence conservation of the epitopes. (PDF) [file ppat.1010924.s008.pdf]

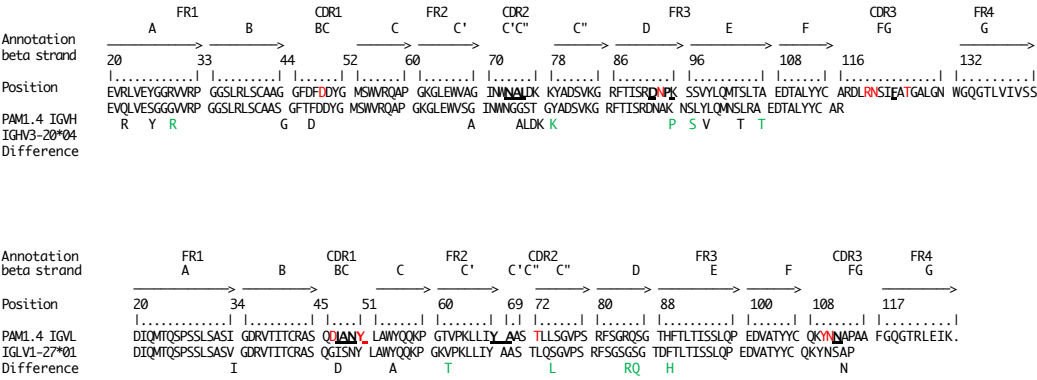

S9 Fig. PAM1.4 sequence annotation.

Supplement: S9 Fig — Alignment and annotation of frame work (FR) and complementarity-determining regions modified from IMTG/quest output. Green letters indicate amino acids with markedly different biochemical properties than the germline sequence. Red letters indicate residues forming hydrogen bonds or electrostatic interactions with VAR2CSA. Bold underlined letters indicate residues stabilizing VAR2CSA binding through hydrophobic interactions. (PDF) [file ppat.1010924.s009.pdf]

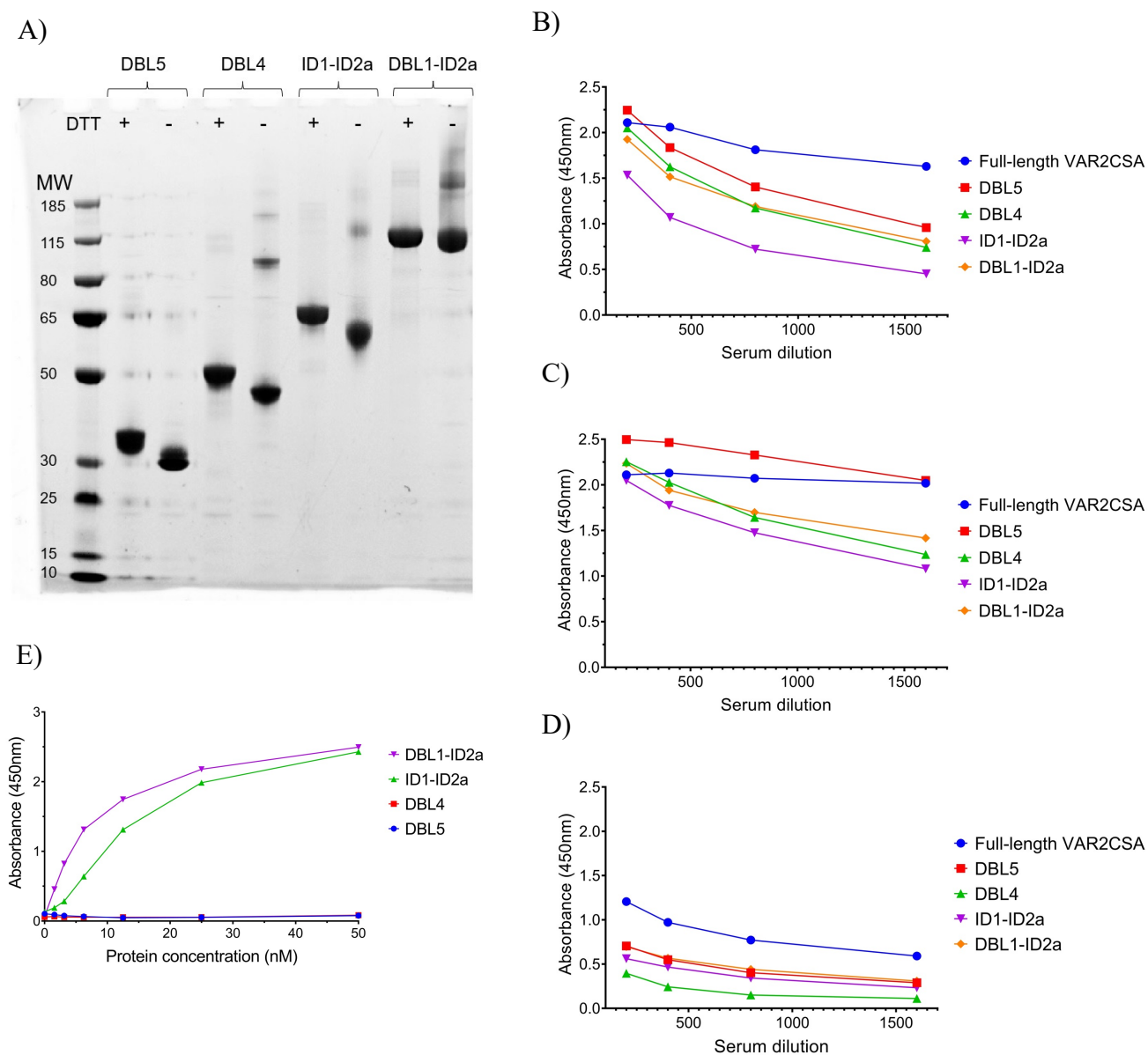

**S10 Fig. Quality control of individual domain proteins of VAR2CSA.**

Supplement: S10 Fig — (A) SDS representation of domain constructs both reduced and non-reduced bands corresponding to the molecular weight. DBL5 (37kDa), DBL4 (53kDa), ID1-ID2a (68kDa), DBL1-ID2a (110kDa). (B-D) ELISA binding of full-length VAR2CSA and individual domains by pools of serum derived from Tanzanian (B) and Ghanaian (C) placental malaria-exposed women and Ghanaian adult males (D), in a dilution series 1:200, 1:400, 1:800 and 1:1600. (E) Decorin CSPG binding assessed by ELISA. Only DBL1-ID2a and ID1-ID2a are showing interaction, as these constructs contain the CSA binding site. (PDF) [file ppat.1010924.s010.pdf]

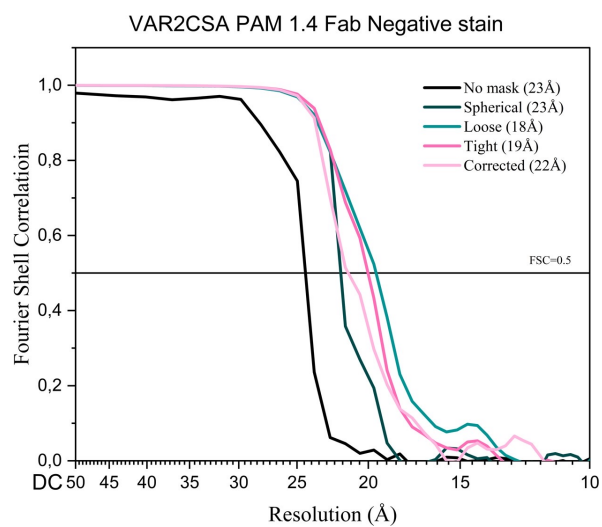

**S11 Fig. GSFSC curve of VAR2CSA PAM 1.4 Fab negative stain map.**

Supplement: S11 Fig — The resolution calculated at 0.5 FSC is 22Å. (PDF) [file ppat.1010924.s011.pdf]
