# Supplementary material for: Cryo-EM reveals the conformational epitope of human monoclonal antibody PAM1.4 broadly reacting with polymorphic malarial protein VAR2CSA
Source: PLoS Pathog. 2022 Nov 16;18(11):e1010924. doi: 10.1371/journal.ppat.1010924 (PMC9668162; doi:10.1371/journal.ppat.1010924)
Supplement: S2 Fig — (a) APO VAR2CSA structure is modelled in the negative stain density of VAR2CSA PAM 1.4 Fab complex to identify and split the density corresponding to the Fab (b) Potential epitopes mapped based on the surface area of the Fab interacting with APO VAR2CSA model structure (c) CryoEM structure of VAR2CSA PAM 1.4 Fab overlayed to verify the density fit and epitopes mapped. (PDF) [file ppat.1010924.s002.pdf]

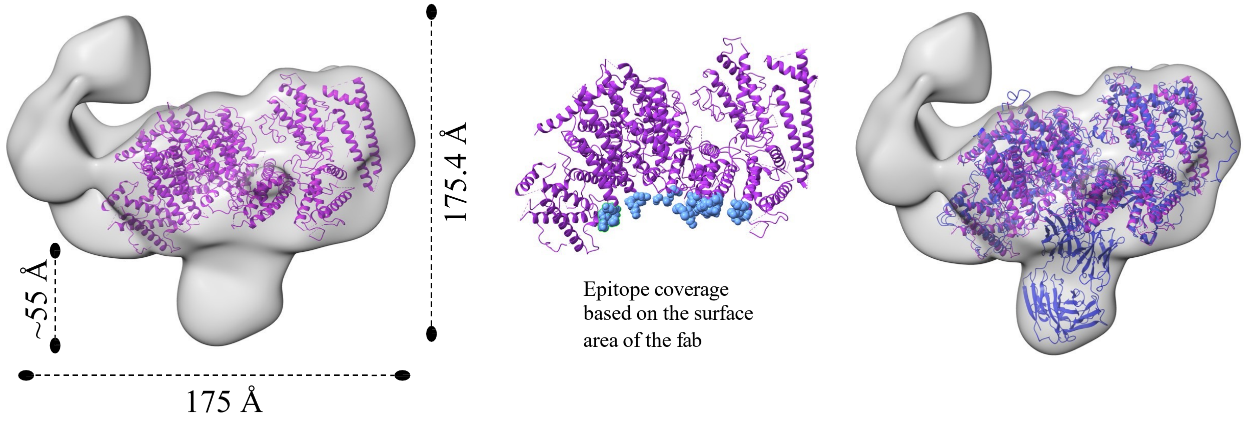

S2 Fig. Model fitting of APO VAR2CSA (PDB ID: 7B52) to map the epitope region of PAM 1.4 Fab as a proof of concept technique to show VAR2CSA Full length is an ideal target for negative stain based polyclonal epitope mapping.
