# Supplementary material for: Cryo-EM reveals the conformational epitope of human monoclonal antibody PAM1.4 broadly reacting with polymorphic malarial protein VAR2CSA
Source: PLoS Pathog. 2022 Nov 16;18(11):e1010924. doi: 10.1371/journal.ppat.1010924 (PMC9668162; doi:10.1371/journal.ppat.1010924)
Supplement: S4 Fig — (PDF) [file ppat.1010924.s004.pdf]

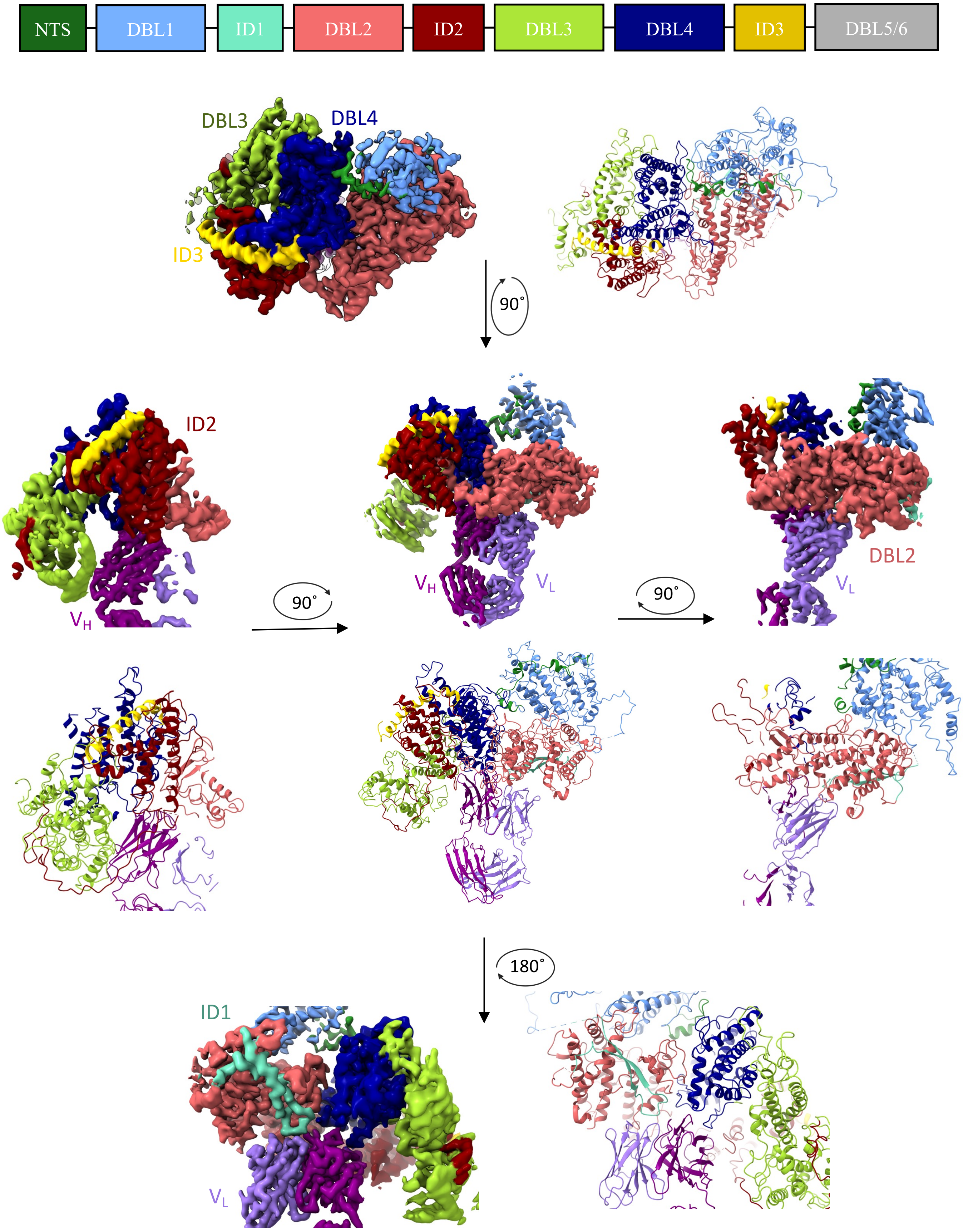

S4 Fig. Different set of views of VAR2CSA + PAM1.4 Fab cryoEM density map and its corresponding refined ribbon model
