# Supplementary material for: Cryo-EM reveals the conformational epitope of human monoclonal antibody PAM1.4 broadly reacting with polymorphic malarial protein VAR2CSA
Source: PLoS Pathog. 2022 Nov 16;18(11):e1010924. doi: 10.1371/journal.ppat.1010924 (PMC9668162; doi:10.1371/journal.ppat.1010924)
Supplement: S5 Fig — Domains are color coded as depicted above. (PDF) [file ppat.1010924.s005.pdf]

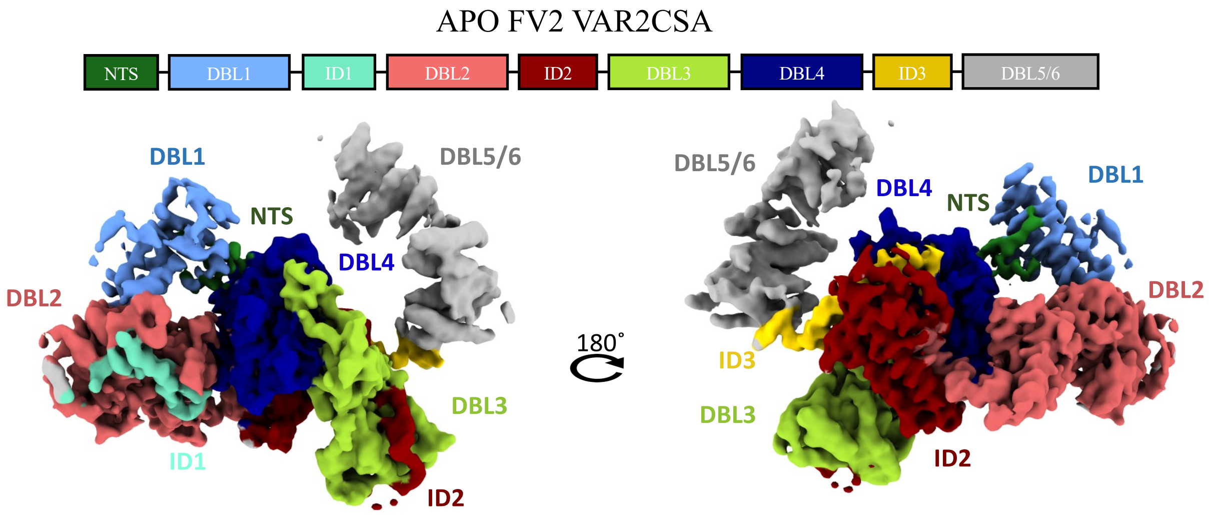

S5 Fig. Molecular architecture of APO-VAR2CSA structure resolved from VAR2CSA unbound to PAM1.4 Fab particles and flipped 180˚ to the right. Domains are color coded as depicted above.
