# Supplementary material for: Cryo-EM reveals the conformational epitope of human monoclonal antibody PAM1.4 broadly reacting with polymorphic malarial protein VAR2CSA
Source: PLoS Pathog. 2022 Nov 16;18(11):e1010924. doi: 10.1371/journal.ppat.1010924 (PMC9668162; doi:10.1371/journal.ppat.1010924)
Supplement: S1 Table — (PDF) [file ppat.1010924.s012.pdf]

S1 Table. CryoEM data collection and refinement statistics for VAR2PAM 1.4

	VAR2PAM1.4	APO VAR2CSA
**PDB**	7Z12	7Z1H
**EMDB**	14438	14446
**Microscope**	Titan Krios	
**Camera**	Falcon III	
**Voltage (kV)**	300	
**Recording mode**	Counting	
**Electron dose (e/Å^2)**	44	
**Defocus range (µM)**	-1 to -2.8	
**Pixel size (Å)**	0.832	
**Micrograph collected**	4927	
**Micrograph used**	4927	
**Total extracted particles**	925142	
**Refined particles**	95911	207129
**Symmetry imposed**	C1	C1
**Map Resolution (Å)**	3.04	3.12
	**Refinement and Validation**	
**R.M.S.D**		
**Bond lengths (Å)**	0.007	0.016
**Bond angles (˚)**	1.038	0.972
**MolProbity score**	2.68	2.36
**Clash score (all atom)**	29.37	19
**Ramachandran plot**		
**Favored (%)**	83.89	88.67
**Allowed (%)**	14.96	10.84
**Rotamer outlier (%)**	1.14	0.06
