# Supplementary material for: Cryo-EM reveals the conformational epitope of human monoclonal antibody PAM1.4 broadly reacting with polymorphic malarial protein VAR2CSA
Source: PLoS Pathog. 2022 Nov 16;18(11):e1010924. doi: 10.1371/journal.ppat.1010924 (PMC9668162; doi:10.1371/journal.ppat.1010924)
Supplement: S2 Table — (PDF) [file ppat.1010924.s013.pdf]

S2 Table. Negative stain data collection parameters for VAR2PAM 1.4

	VAR2PAM1.4	APO VAR2CSA
**Microscope**	TALOS Arctica 200kV	
**Camera**	Falcon III	
**Voltage (kV)**	200	
**Recording mode**	Counting	
**Electron dose (e/Å²)**	43.5	
**Defocus range (µM)**	-0.6 to -1.2	
**Pixel size (Å)**	1.54	
**Micrograph collected**	2347	
**Micrograph used**	2345	
**Total extracted particles**	188434	
**Refined particles**	46532	86877
**Symmetry imposed**	C1	C1
**Map Resolution (Å)**	22	19
