# Supplementary material for: Cryo-EM reveals the conformational epitope of human monoclonal antibody PAM1.4 broadly reacting with polymorphic malarial protein VAR2CSA
Source: PLoS Pathog. 2022 Nov 16;18(11):e1010924. doi: 10.1371/journal.ppat.1010924 (PMC9668162; doi:10.1371/journal.ppat.1010924)
Supplement: Attachment — Submitted filename: Response_to_reviewers.pdf [file ppat.1010924.s014.pdf]

Dear Dr. wang,

Thank you very much for submitting your manuscript "Cryo-EM reveals the conformational epitope of human monoclonal antibody PAM1.4 broadly reacting with polymorphic malarial protein VAR2CSA" for consideration at PLOS Pathogens. As with all papers reviewed by the journal, your manuscript was reviewed by members of the editorial board and by several independent reviewers. In light of the reviews (below this email), we would like to invite the resubmission of a significantly revised version that takes into account the reviewers' comments.

The reviewers have raised a number of issues that need to be addressed before your paper would be considered acceptable for publication in PLOS Pathogens. In particular, please consider their comments on the **need for additional experimental controls**, and more clearly **address the potential protective functions of the MAb**. Please comment on the **ways in which these findings can inform vaccine development**, as noted by Reviewer 2, since the MAb does not have the desired property of inhibition of binding to CS.

Reply:

Dear editors,

Thank you very much for considering our manuscript for publication. In the resubmitted manuscript we have positively addressed all comments made by the reviewers. We have provided the requested experimental controls and paid particular attention to clarify the potential protective functions of the mAb and its potential for informing vaccine development, as highlighted by you.

As per reviewers' request, we have added reactivity of polyclonal serum of malaria exposed donors to VAR2CSA recombinant proteins, For this reason additional authors (Michael F. Ofori, Ghana, Daniel T.R. Minja, Tanzania, Christentze Schmieglow, Denmark, and Sebastian B. Christensen, Denmark), who conducted serum collections and reactivity Elisa against VAR2CSA fragments have been included. We hope our responses to the questions raised are satisfactory.

Kind regards,

Kaituo Wang et al.

Letter: Detailed list of responses to the review comments

Part I – Summary

Reviewer #1: Understanding the molecular and structural basis for broadly inhibitory antibodies to VAR2CSA would be critical in the development of a broadly protective placental malaria vaccine. Here the authors use cryo-EM to evaluate the binding site of the broadly reactive antibody, PAM1.4 Fab, and found that it binds a highly conserved conformational epitope involving several domains of the core VAR2CSA structure. The authors suggest that while broadly reactive, that this antibody does not functionally block the interaction of VAR2CSA and CS.

The study employs a useful technique for resolving protein structures – cryo-EM – to generate molecular details of the interaction of PAM 1.4. This data is compelling and adds additional details to the structure-based approaches for VAR2CSA vaccine target design, in addition to further validating the utility of negative stain and cryo-EM approaches for resolving protein structures in malaria, and specifically for VAR2CSA. The structural data is a strength of the paper. Some of the binding and functional experiments could be further strengthened and appropriate controls included so that the conclusions are truly justified by the data presented.

The paper is clear and well-written. I hope that the suggestions below will help the authors improve the manuscript.

Reviewer #2: This study has resolved the cryoEM structure of full length VAR2CSA ectodomain in complex with a broadly neutralizing antibody (Fab of PAM 1.4). The work is for the most part presented clearly and carefully performed. While the structure reveals the epitope of PAM 1.4, the study lacks defining a mechanism of action of the monoclonal antibody. This is further detailed in the "Major Issues" section of the review. Overall, this is an interesting structural study and with some revisions could be further strengthened.

> *REPLY*: We thank the reviewers for their positive comments and have addressed the main points raised as detailed in the answers below.

Part II – Major Issues: Key Experiments Required for Acceptance

Reviewer #1: Results:

1. Fig 1A. Line 91-93: Validation of the expressed VAR2CSA domains would be important especially when showing no direct binding. Can positive binding be demonstrated with polyclonal polygravid immune serum or vaccinated IgG? This would be important to demonstrate the protein domains are properly folded. This will be especially important to demonstrate given the conclusions stated in Lines 114-115. To make such claims, the authors should demonstrate that the protein domains themselves are properly folded and able to be bound by antibodies previously shown to react to these domains. This will make the contrast w with PAM 1.4 more dramatic and convincing.

REPLY: We agree that the quality control data on the proteins should be provided. We have added supplementary data of SDS gels to show protein purity, and for proteins spanning the CSA binding site, we have added data showing positive CSA binding to provide support for the protein´s structural and functional integrity. Immune-serum recognition of the proteins using pooled serum from multigravida women from Tanzania and Ghana and male serum is included as well. Data are shown in new supplementary figure S10.

2. Fig 1B. Line 93-95: Can the authors verify that there is strain specific binding by flow of the other monoclonal antibodies? (Thus PAM1.4 Fab, in contrast to other antibodies, is truly pan-strain binding). A negative control condition (for example, non-CSA panned parasites of the same strains that should show decreased VAR2CSA expression and decreased binding, or DTT and Trypsin treatment to allow cleavage of VAR2CSA from the surface of the IEs), should be included to show that the positive binding is clearly specific and reversible. The flow-through from the mAb-bead-column preparations would also be a helpful control. Here, the authors used beads coupled with the mAb PAM1.4 to pre-select for PAM1.4 binding late trophozoites and schizonts. It would be helpful to see what percentage of VAR2CSA expressing IEs are truly recognized by PAM 1.4.

REPLY: The unique breadth of PAM1.4 reactivity was imprecisely described. Indeed, among the eight mAbs originally identified in Barfod et al Mol Microbiol 2007, all reacted with several natively expressed VAR2CSA variants, but only PAM1.4 reacted to all 5 of 5 variants tested. PAM1.4 has since been used in several independent investigations (examples include Sampaio et al. Infection and Immunity 2017, Doritchamou et al Communications Biology 2019), and appears to react with essentially all VAR2CSA variants. To clarify, we have changed lines 104-105 to

"Of these, five exhibit reactivity with several (but not all) sequence diverse natively expressed VAR2CSA variants, whereas one, PAM1.4, appears to recognize all (or at least most) native VAR2CSA variants [26-28]"

Also line 224 was changed to "Broadly reactive human monoclonal antibodies to VAR2CSA are likely to be common among women naturally exposed to VAR2CSA….".

PAM1.4 specificity for VAR2CSA parasites was shown in Barfod et al 2007, but for the specific experiments presented here, we have now included, in new Figure 1B, the negative control data of PAM1.4 reactivity to non-VAR2CSA 3D7 IE, as well as IgG isotype controls, i.e. malaria-naïve human IgG reactivity to the assessed parasites. While IEs are pre-selected using PAM1.4, the PAM1.4 flow cytometry staining is conducted on IEs expanded in culture following selection. As shown in the figure the percentages of IEs stained by PAM1.4 is very high (80-90% of IEs). It is not possible

to assess to which degree the proportion of PAM1.4 negative cells are expressing other PfEMP1 or indeed expressing VAR2CSA at a lower level or in a PAM1.4-inaccessible surface presentation. However, PAM1.4 has been shown to bind the majority of IEs expanded following selection for CSA binding (e.g in Doritchamou et al., Communications Biology 2019), suggesting PAM1.4 is specific VAR2CSA and native VAR2CSA can be targeted by PAM1.4. Conversely, selection of IEs with PAM1.4 leads to increased VAR2CSA expression (Soerli et al. Parasite Immunol, 2009).

3. Figure S1. It would be ideal if the authors could provide flow data using an isotype matched unrelated antibody as the negative control to rule out non-specific binding.

REPLY: This has now been included. See response above.

4. Figure S7. A major finding of the paper, and a point of emphasis of the authors in the introduction and discussion, this data should be moved to the main text, but would require additional replicates. The error bars are a bit wide for the two replicates and appears that PAM1.4 inhibits more significantly in one experiment compared to the second. The authors should describe the characteristics of each antibody in the legend: Fab = PAM 1.4 Fab and IgG = PAM 1.4 total IgG?

NOTE: This comment is further detailed in clarification 2, reviewer 1:

Reviewer #2: What is shown is that the antibody binds some conserved residues (up to 3/4 of VAR2CSA variants) that are distant from the binding site with CSA. The failure of PAM 1.4 to inhibit VAR2CSA binding to CSA is also shown (Figure S6). It would have been good for the authors to further examine mechanism. Does the PAM 1.4 IgG interact with complement, monocytes or Fc-gamma-Rs to carry out a broadly protective antibody-mediated activity? Or does PAM 1.4 IgG just bind a broad range of VAR2CSA variants without any correlates of protective immunity. These data are needed for the various claims in the manuscript around how this structural work could benefit future vaccine design. Should the above data not be available then the tone of the manuscript needs to be modified throughout. Some minor comments are also provided below.

REPLY: In early studies (Barfod et al., 2007 and 2010), PAM1.4 was isolated and produced from EBV-immortalized memory B cells. Thus, the Fc characteristics of the native antibody cannot be fully accounted for. However, the PAM1.4 from such B-cells was shown to opsonize IEs for phagocytosis by THP-1 cells (Barfod et al., J Immunol 2010). Later, Larsen et al., Front. Immunol 2019 cloned the PAM1.4 VH and VL chains into commonly used vectors (Tiller et al J Immunol Methods 2008) encoding gamma1 and kappa constant regions and further introduced Fc mutations enhancing complement activation. These recombinant versions of PAM1.4 were able to activate complement on recombinant VAR2CSA but *not* on native or immobilized VAR2CSA PfEMP1, indicating an unknown mechanism of immune escape by VAR2CSA.

Most recently, Larsen et al Nature Comm 2021 produced two fucosylation variants of recombinant PAM1.4. The variants bound recombinant full length VAR2CSA equally well, but only the afucosylated PAM1.4 induced marked NK-cell degranulation. These results mirrored observations that naturally acquired VAR2CSA-specific IgG is strongly afucosylated and efficiently induce FcγRIIIa-dependent natural killer (NK) cell degranulation.

Afucosylated IgG appears to be specifically induced by foreign antigens expressed on self-membrane, such as those of enveloped viruses and PfEMP1 (Larsen et al. Science 371, eabc8378, 2021; Larsen et al. Nat Commun, 2021). Afucosylation increases the ability to elicit FcγRIII-dependent effector responses such as NK-cell ADCC (Bruggeman et al. J Immunol, 2017).

In contrast, VAR2CSA protein subunit vaccination induces fully fucosylated IgG, which has profound implications for future vaccine design (Larsen et al. Nat Commun, 2021). Fc-modified versions of PAM1.4 can be used to benchmark the effects of future vaccines.

We modified the manuscript to clarify the potential use of PAM1.4 in the context of vaccine development while emphasizing that the structural work presented here does not in itself provide guidance to new Fc-directed vaccine design. We have omitted line 230 of the discussion and rearranged the discussion including adding following paragraph.

Line 231: "…. Thus, it is possible that broadly neutralizing VAR2CSA antibodies function by direct competition with CS at the CS binding site or sterically inhibiting upon binding near the CS epitopes. CS-binding inhibition is thought to be a key mechanism of acquired immunity to placental malaria [36]. However, the relative importance of neutralization and opsonization remains unknown. Indeed, acquired antibodies to VAR2CSA are mainly cytophilic [37] and importantly, are markedly afucosylated (Larsen et al. Nat Commun, 2021). This trait is shared with IgG elicited by other host membrane-associated proteins such as antigens of enveloped virus (Larsen et al. Science, 2021) . Thus, Fc-modulated versions of PAM1.4 and other broadly reactive but non-inhibitory antibodies may serve as useful tools for benchmarking vaccine-elicited cellular-mediated immunity to placental malaria.
The structural resolution of VAR2CSA has raised hopes that novel immunogens and vaccine strategies for boosting cross-reactive responses can be designed [38]. However, for this to succeed, identification and characterization of new broadly reactive, and ideally, CS-binding inhibitory VAR2CSA antibodies is likely needed……"

Part III – Minor Issues: Editorial and Data Presentation Modifications

Reviewer #1: Suggestions:

1. The manuscript represents a very interesting finding and an important use of cryo-EM to

help resolve structural interactions between an interesting mAb and its binding domain on VAR2CSA. However, the manuscript feels brief (almost as if it has been written in the format of a short report). Some of the additional data presented in supplemental (for example S3, S4, S5, S6, S7) could be moved to the main text as panels within the existing figures to provide more detail and clarity.

> *REPLY*: We have moved (previously Figure S4 and S6) into the main manuscript (as Figure 3A and 3B) to show PAM1.4 Fab binding epitopes are distant from CS binding epitopes of VAR2CSA and that there is no conformational change in VAR2CSA upon PAM1.4 binding.

Clarifications:

2. Line 81-82 – The authors claim, "whereas the early data indicating that PAM1.4 was also able to inhibit IE adhesion to CS have not been subsequently verified." Please cite the source of this early data (reference if published) and indicate why there is controversy as to whether this data is credible, or in need of further verification. Do the authors mean that it has never been reproduced or independently verified? In the discussion (Lines 180-181), the authors mention that there is unpublished data from several groups. Can these data be listed as "personal communication" or "unpublished" and attributed to the groups/authors?

> *REPLY*: We understand that the figure S6 (Previously S7) data could appear key or controversial. However, lack of potent CSA-IE binding inhibitory effect of PAM1.4 has previously been concluded without publishing of data, by the two University of Copenhagen-based teams collaborating on this publication (Morten Nielsen, and Lars Hviid teams) as well as by Patrick Duffy´s team at NIH, who performed the initial studies suggesting inhibitory activity of the mAb. In reflection of this, Duffy et al state in Renn et al Commun Biol. 2021, as accepted knowledge albeit without providing experimental support that: "Importantly, while PAM1.4 recognizes an epitope displayed by full-length VAR2CSA, PAM1.4 does not exhibit anti-adhesion activity". It has not been possible for us to unravel what caused the discrepancy, but the statement aligns with the shared knowledge in the field. Based on this and the molecular resolution of the CSA-VAR2CSA complex and the PAM1.4 epitope results presented here, we feel the data in S7 is sufficient and best kept as supplementary data. To clarify in the Manuscript, previous lines 81-82 (Now 106) (introduction) and 180-181 (now 207) (discussion) have been changed and now include published and unpublished references.
> The previous figure S7 (now Figure S6) legend has been corrected as suggested.
>
> Line 106 changed to: PAM1.4 can efficiently opsonize IEs for Fc receptor-dependent killing in vitro [18, 29], whereas unpublished studies revoking early data has led to the consensus that PAM1.4 is unable to inhibit IE adhesion to CS [Renn et al., 2021].

Line 207 changed to: However, unpublished research by our and other research teams led to the consensus notion that PAM1.4 is cross-reactive but not neutralizing [Renn at al., Commun Biology, 2021]

3. Lines 142-147: Presented under results, it is unclear if the authors are referring to their own alignments or merely citing previously published studies of VAR2CSA diversity. It seems that the later is the case, which is fine, but if so this should be moved into the discussion. If the authors have generated sequence alignments themselves to look at diversity of the PAM 1.4 binding epitopes (perhaps of the specific strains they used in their binding assays), this data should be presented in the paper as a figure allowing the reader to visualize the highly conserved versus slighly more variable epitopes included in the mAb binding region.

REPLY: The source and use of the VAR2CSA alignment was indeed unclear. We have used our previously published alignment of 3737 sequences extracted from the var database, varDB PF3K and annotated with DBL domains. A precise reference to the logo is now given. The alignment includes sequences of the cryoEM-resolved variant presented here. The presentation of sequence diversity of interacting residues is best suited for the results section. We have clarified where the reader can find the published sequence alignment and for each amino acid position of the FCR3 VAR2CSA variant described in text and figures, we have in the figure legends, indicated the position in the corresponding position in said alignment.

4. Lines 205-209: The authors' final conclusion is that PAM 1.4 is broadly reactive, but not neutralizing. However, in the introduction, the authors mention that PAM1.4 can be protective via the mechanism of opsonization of IEs for Fc receptor-dependent killing in vitro, citing previous studies. The authors should comment on the relative contributions of these two mechanisms in the context of protection from placental malaria in the discussion.

REPLY: The Manuscript and discussion have been altered to cover this, as described in answer to reviewer 2 above.

Some suggestions for typos/grammar:
5. Line 55 – "VAR2CA variants" - VAR2CSA variants

REPLY: corrected

6. Throughout: even though the first mention of a term is correctly followed by an abbreviation, there are some abbreviations that feel unnecessary and are a little distracting.

The authors might consider leaving the unabbreviated term, especially for shorter phrases (such as IEs – infected erythrocytes; PM – placental malaria, etc). This is merely a stylistic suggestion.

REPLY: we agree and have corrected accordingly

Reviewer #2: Abstract, "High-resolution data shows..." the structure was determined at a global resolution of around 3Å and at around 2.5 Å at the antibody:antigen interface. This does not represent high resolution, but actually medium to low resolution data. Please modify here and and other references to high resolution in the manuscript

REPLY: Corrected.

Lines 94 - 96 (Fig 1B and S1) describes flow cytometry of PAM1.4 binding to VAR2CSA variant infected RBCs. However, there is no suitable negative controls provided (a non-VAR2CSA parasite; isotype control - or at least this is not shown in the figure captions).

REPLY: This has now been included, see point 2 reviewer #1.

Fig 1B. These ELISA plots are single lines without information about variation. How many replicates were performed and if sufficient repeats then appropriate errors should be included. If only duplicates it is best practice to plot both data points against the lines of best fit.

REPLY: Error bars of 2x duplicate determinations have now been added.

Fig 2 is fine to give an overall representation of the cryoEM structure. It would be good to also include here or in a separate figure a cleaner set of views of the refined structural model.

REPLY: We have made a new supplementary figure (Fig. S4) showing different views of the structure for better clarity.

Fig 3 shows interactions but much of the details are hard to see. In particular text sizes are particularly small across this entire figure.

REPLY: We have separated the previous figure 3 into two figures (Figure 4 and Figure 5) with higher magnification for better visualization of all the information.
